# Comparative analysis of techniques for open-window thoracostomy

**DOI:** 10.36416/1806-3756/e20250311

**Published:** 2026-03-05

**Authors:** Karine Letícia Ferreira Machado da Costa, Gustavo Henrique Gadelha de Medeiros, Nabor Bezerra de Moura

**Affiliations:** 1. Centro Universitário UNINOVAFAPI, Teresina (PI) Brasil.; 2. Hospital Getulio Vargas, Teresina (PI) Brasil.; 3. Universidade Federal do Piaui, Teresina (PI) Brasil.

## TO THE EDITOR:

Pleural empyema is a collection of infectious material in the pleural cavity, being usually secondary to pneumonia.^1^ Pleural empyema as a complication of parapneumonic pleural effusion has a mortality rate of 6-10%, with an estimated incidence of 14,000-20,000 cases per year in Brazil.^2^

Failure of less invasive treatment modalities or delayed treatment requires surgical intervention, involving pleural decortication or thoracostomy. The Eloesser procedure, also known as pleurostomy or open-window thoracostomy (OWT), is indicated for the treatment of chronic pleural empyema. The objective of OWT is to drain purulent secretion from the pleural cavity and allow lung re-expansion.^3^ ^,^ ^4^ However, because OWT has undergone various changes over the years, there is currently no consensus regarding the procedure that would yield the best outcome. The choice of OWT technique is based on institutional practice and local protocols based on experience.^3^ ^,^ ^4^ Therefore, the objective of the present study was to determine the best technique for OWT on the basis of time to surgical wound closure and recurrence rates. 

We performed a retrospective observational study at a tertiary public hospital in the city of Teresina, Brazil. The *Hospital Getulio Vargas* has 384 beds, of which 40 are ICU beds. Data were collected from medical records of patients undergoing OWT from January of 2016 to December of 2018. Patients who had no electronic medical records or who did not return to the outpatient clinic were excluded. 

We collected data on the following variables: affected side, type of incision, number of resected ribs, and suture size. Absorbable synthetic sutures were used in all patients. 

Data were processed with Microsoft Excel 2019. The results were expressed in proportions and were tabulated, with time to surgical wound closure and recurrence rates being compared in terms of sex, age, affected side, type of incision, suture size, and number of resected ribs. The chi-square test was used for qualitative variables, whereas the two-tailed t-test was used for quantitative variables, with a confidence level of 95%. All statistical analyses were performed with the IBM SPSS Statistics software package, version 29.0 (IBM Corporation, Armonk, NY, USA). 

The present study was conducted in accordance with the Declaration of Helsinki and was approved by the Research Ethics Committee of *Centro Universitário UNINOVAFAPI*, located in the city of Teresina, Brazil (Protocol no. CAAE 09065019.2.3001.5613). 

We collected data from the medical records of 31 patients who underwent OWT at our institution in the period between January of 2016 and December of 2018. The mean age was 43.68 ± 17.46 years. Thirty patients returned for postoperative evaluation, and one patient died during follow-up. For some of the study variables, the sum may be smaller than the total sample because of data missing from the medical records. Other data are summarized in Table 1. 

**Table 1:** Characteristics of open-window thoracostomy procedures performed at our institution between 2016 and 2018.

Variable		n	%
Sex	Male	23	74.8%
	Female	8	25.2%
Side	Right	15	51.7%
	Left	14	48.3%
Incision	Longitudinal	11	35.5%
	Transverse	7	22.6%
Suture size	1	15	48.4%
	0	6	19.4%
	2-0	1	3.2%
No. of resected ribs	1	11	35.5%
	2	15	48.4%

A total of 5 patients (16.7%) experienced recurrence, and 25 (83.3%) did not. Time to surgical wound closure was ≤ 90 days in 5 patients (17.2%); 91-180 days in 6 (20.7%); 181-365 days in 9 (31.0%); and > 365 days in 9 (31.0%). 

With regard to sex, 18 male patients had no recurrence (60.0%), whereas 7 female patients had no recurrence (23.3%; p = 0.177). The mean age of the patients who experienced recurrence was 44.8 ± 11.65 years, and that of those who did not was 44 ± 18.2 years (p = 0.903). Of the patients with right hemithorax involvement, 12 (44.4%) experienced no recurrence. Of those with left hemithorax involvement, 10 (37.0%) experienced no recurrence (p = 0.557). 

As can be seen in Table 2, type of incision, suture size, and the number of resected ribs were not associated with significant differences in recurrence rates. 

**Table 2:** Recurrence of empyema and time to surgical wound closure for different open-window thoracostomy techniques performed in patients undergoing surgery at our institution between 2016 and 2018.

Variable		Recurrence					Time to closure, days								
		Yes		No		p	16-90		91-180		181-365		> 365		p
		n	%	n	%		n	%	n	%	n	%	n	%	
Incision	Longitudinal	1	6.2%	9	56.2%	0.696	0	0.0%	3	17.6%	5	29.4%	2	11.8%	0.302
	Transverse	1	6.2%	5	31.2%		1	5.9%	2	11.8%	1	5.9%	3	17.6%	
Suture size	1	2	10.0%	11	55.0%	0.550	2	9.5%	3	14.3%	6	28.6%	3	14.3%	0.665
	0	0	0.0%	6	30.0%		0	0.0%	2	9.5%	2	9.5%	2	9.5%	
	2-0	0	0.0%	1	5.0%		0	0.0%	1	4.8%	0	0.0%	0	0.0%	
No. of resected ribs	1	0	0.0%	10	41.7%	0.118	1	4.2%	4	16.7%	4	16.7%	1	4.2%	0.125
	2	3	12.5%	11	45.8%		1	4.2%	2	8.3%	3	12.5%	8	33.3%	

In most (27.6%) of the male patients, time to surgical wound closure was 181-365 days, whereas, in 3 female patients, time to surgical wound closure was > 365 days (p = 0.689). Regarding the affected hemithorax, time to surgical wound closure was ≤ 90 days in 4 of the patients with right side involvement (14.8%); 91-180 days in 5 (18.5%); 181-365 days in 4 (14.8%); and > 365 days in 2 (7.4%). For those with left side involvement, time to surgical wound closure was ≤ 90 days, in 1 patient (3.7%); 91-180 days, in 1 (3.7%); 181-365 days, in 3 (11.1%); and > 365 days, in 7 (25.9%; p = 0.068). 

As can be seen in Table 2, incision type, suture size, and the number of resected ribs were not associated with significant differences in healing time. 

OWT consists of creating a window in the chest wall to allow drainage of pleural fluid and lung re-expansion. However, there have been many changes in OWT techniques over the years. Nevertheless, we found no significant differences in outcomes across techniques.^5^

The treatment of empyema includes drainage of infected pleural fluid, antibiotics, control of underlying factors, intrapleural enzyme therapy, and surgery if the disease becomes chronic. The **R**enal (urea level), **A**ge, **P**urulence (of pleural fluid), **I**nfection source, and **D**ietary (albumin level) score is a validated risk prediction score and aids in patient management based on risk factors. With improved antibiotic therapy and advances in minimally invasive thoracic surgery, the use of procedures such as OWT has decreased. Nevertheless, there are cases in which OWT cannot be avoided.^4^ ^,^ ^6^ ^,^ ^7^

Although pleural infection is predominantly observed in the pediatric and elderly populations, an increase in incidence has been observed among young adults. Previous studies have shown that the mean age of patients hospitalized for empyema ranges from 41.86 years to 68 years, with the majority (74.6-76.2%) of patients being male.^1^ ^,^ ^8^ In our sample, the mean age of the patients was 43.68 years and 74.2% were male, findings that are consistent with the literature and show an increased incidence of pleural involvement in younger populations. 

Many surgeons opt for resecting two to four ribs when performing an OWT.^9^ ^,^ ^10^ Although a higher number of resected ribs can facilitate the closure of a bronchopleural fistula, smaller windows appear to increase the likelihood of closure.^5^ ^,^ ^8^ One study reported a period of 8-12 months for complete closure of the thoracic cavity.^10^ We found a trend toward longer closure times when larger windows were made, although the difference was not significant. On the other hand, larger windows with more than one resected rib showed no less recurrence than did smaller windows. This trend toward smaller incisions is supported by the recent literature.^8^ ^,^ ^9^

Ideally, an OWT incision should allow the best closure of the cavity after proper pleural clearance, thus avoiding recurrence.^10^ However, we found no relationship between the type of incision and the postoperative results. 

The present study has limitations such as its retrospective nature, which made it impossible to recover data on all of the study variables. In addition, the number of procedures analyzed was not very large, which may have limited the analysis. On the other hand, this is the first study to report on the suture size for a pleurocutaneous anastomosis and the postoperative results of OWT. Larger, prospective studies are needed to corroborate our findings. 

The present study showed no superiority of any specific OWT technique. Therefore, the choice of OWT technique can be based on surgeon experience. Smaller, less invasive surgeries can be safely performed. 
